# Pressure‐Induced Emission Enhancement of Multi‐Resonance o‐Carborane Derivatives via Exciton‒Vibration Coupling Suppression

**DOI:** 10.1002/advs.202411765

**Published:** 2025-01-27

**Authors:** Zening Li, Qing Zhang, Fangxiang Sun, Chunyan Lv, Xinmiao Meng, Yu Hu, Dongqian Xu, Chengjian Li, Lei Li, Kai Wang, Yujian Zhang

**Affiliations:** ^1^ Key Laboratory of the Ministry of Education for Advanced Catalysis Materials Department of Chemistry Zhejiang Normal University Yingbin Road No.688 Jinhua 321004 P. R. China; ^2^ Department of Materials Chemistry Huzhou University East 2nd Ring Rd. No. 759 Huzhou 313000 P. R. China; ^3^ School of Physics Science and Information Technology Liaocheng University Hunan Road No. 1 Liaocheng 252000 P. R. China

**Keywords:** exciton‒vibration couplings, fluorescence, multiple resonance, o‐carborane

## Abstract

Polycyclic multiple resonance (MR) molecules reveal narrowband emission, making them very promising emitters for high color purity display. Nevertheless, they still have challenges such as aggregation‐induced emission quenching and spectral broadening. Overcoming these obstacles requires an in‐depth understanding of the correlations among the alterations in their geometries, packing structures, and molecular vibrations and their corresponding changes in their photoluminescence (PL) properties. Herein, it is demonstrated that high‐pressure infrared, UV−visible absorption, and fluorescence spectroscopies can be combined with computational results to elucidate the influence of the subtle structural variations on the exciton‒vibration couplings and their PL properties. An ortho‐carborane‐decorated MR emitter (BNC) is a piezochromic molecule and exhibits emission enhancement under high pressure. A thorough analysis of the in situ experimental measurements and calculated results reveals that the pressure‐induced changes in the exciton binding energy and exciton‒vibration couplings are responsible for the unusual piezochromism. This research provides insights into the structure‒fluorescence relationship and potential for high‐pressure techniques to optimize MR materials for advanced organic light‐emitting diodes (OLEDs) applications.

## Graphical abstract

A polycyclic multiple resonance architecture with icosahedral boron clusters is developed and its piezochromism, exciton binding energy and exciton–vibrational couplings are fully investigated. During compression, the multi‐resonance emitter exhibits the emission enhancement due to the decrease in exciton–vibrational couplings.

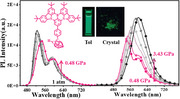

## 1 Introduction

Organic light‐emitting diodes (OLEDs) are widely used in mobile devices such as smartphones, flat panel displays, and wearables. They are lightweight, ultrathin, wide‐angle, and flexible and can provide an excellent viewing experience. As the color gamut standard for next‐generation displays, the ITU‐R BT.2020 standard requires the full width at half‐maximum (FWHM) of the monochromatic red, green, and blue (RGB) spectra to be less than 30 nm.^[^ ^1^, ^2^, ^3^, ^4^ ^]^ Conventional organic emitters, such as perylene and coronene, have very rigid polycyclic aromatic hydrocarbons and commonly exhibit relatively small structural relaxations with small Stokes shifts. However, the “breath” of their excited state is produced mainly by the vigorous stretching vibration of the molecular bond, producing multiple fine vibration emissions with large FWHM values.^[^ ^5^, ^6^, ^7^ ^]^ In 2016, Hatakeyama et al. developed a N/B‐doped rigid heterocycle,^[^ ^8^ ^]^ in which the frontier orbitals were effectively separated. This so‐called multiple resonance (MR) effect could attain short‐range charge transfer transition and result in a small FWHM value of the EL spectrum.^[^ ^2^, ^9^, ^10^, ^11^, ^12^, ^13^ ^]^ Nevertheless, MR emitters have two key issues including the evident aggregation‐caused photoluminescence (PL) quenching and spectral broadening.^[^ ^14^, ^15^, ^16^, ^17^ ^]^ For example, Monkman et al. reported that even at extremely low doping concentrations, MR emitters could exhibit a shoulder peak at a longer wavelength, resulting in large FWHM values and lower‐than‐expected device performance.^[^ ^17^, ^18^ ^]^

The FWHM value is dependent on the structural relaxation and vibration coupling of the excited states. Molecular vibration is closely associated with the optical phonons, as described by Marcus theory. This connection implies that the interaction between the excitons and optical phonons can manifest as exciton‐vibration coupling. To quantify this interaction, the FWHM of the PL spectra can be fitted to the following equation (Equation (1)):^[^ ^19^ ^]^

(1) $$\Gamma \left(T\right)=2.36\sqrt{S}\hbar \omega \sqrt{\coth\left(\frac{\hbar \omega }{2\kappa_{B}T}\right)}$$

Here, *Γ*(*T*) represents the FWHM at temperature *T*, and *S* and *ħω* denote the Huang–Rhys factor and the effective vibrational energy coupled in the emission spectra, respectively. The FWHM of PL spectra generally increases along with the molecular vibration due to the strong interaction between the excitons and optical phonons. In the context of aggregation‐induced PL quenching, the photoluminescence quantum yields (PLQYs) of organic emitters are determined by the nonradiative decay rate constant (*k*_nr_). According to the band‐gap law, *k*_nr_ can be simply expressed as follows:^[^ ^20^, ^21^ ^]^

(2) $$k_{\mathrm{nr}}\propto V^{2}{\left(4\pi \lambda_{S}\kappa_{B}T\right)}^{-0.5}\sum_{j=0}^{\infty }\frac{e^{-s}S^{j}}{j!}\exp\left[-\frac{{\left(E_{g}-j\hbar \bar{\omega }-\lambda_{s}\right)}^{2}}{4\lambda_{s}\kappa_{B}T}\right]$$

where ϖ is the mean frequency of high‐frequency vibration with additional parameters detailed in the Supporting Information. This formula shows a significant impact of molecular vibrations, highlighting the influence of exciton‒vibration couplings on *k*_nr_. Notably, the high‐frequency vibration is attributed to low‐mass vibration and is derived from either the hydrogen‒carbon bonds or carbon‒carbon bonds of the organic emitters. Clearly, to fully understand and effectively address the intractable issues of the severe aggregation‐induced PL quenching and spectral broadening, thoroughly elucidating the exciton‒vibration coupling behavior of MR emitters in the solid state is essential. However, the manipulation and experimental detection of free twisting and stretching vibrations of the aromatic skeletons, closely related to exciton–vibration couplings, is greatly challenging.

**Figure 1:**
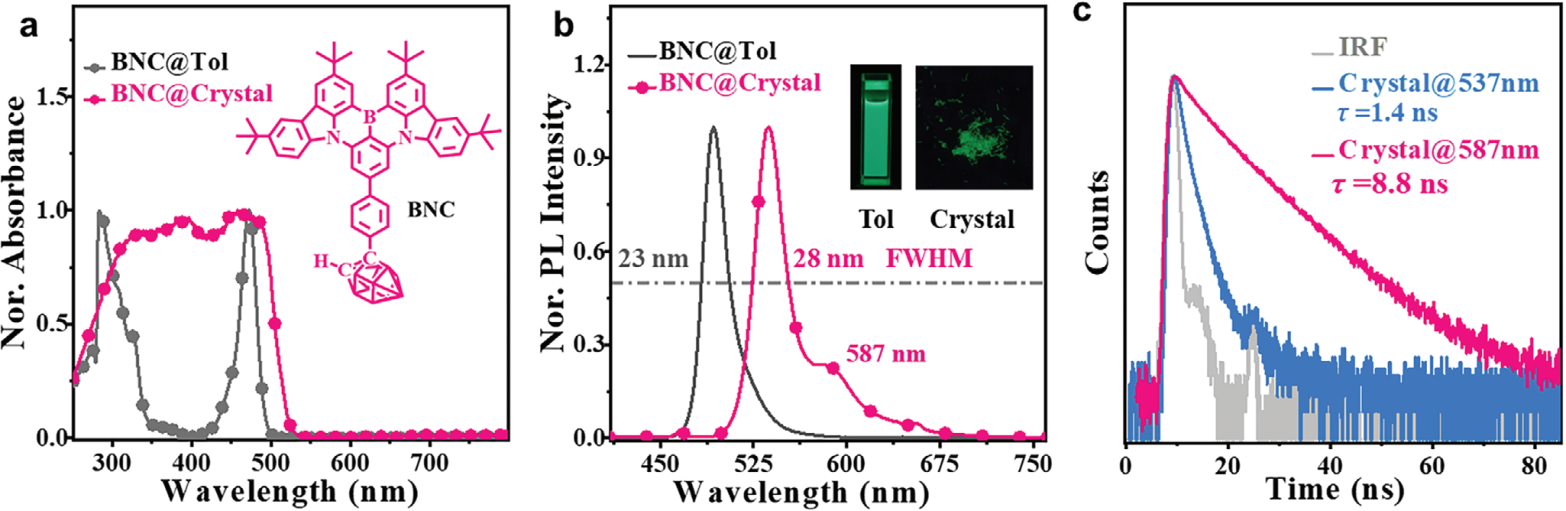
a) UV–visible absorption spectra of BNC in toluene (1 × 10^−5^ m) and in the crystalline state. Inset: Chemical structures of BNC; inset: molecular structure of BNC. b) Steady‐state fluorescence of BNC in toluene (1 × 10^−5^ m) and the crystalline state; inset: corresponding photographs of BNCs collected under a 365 nm UV lamp. c) Transient PL decay curves of the BNC crystal.

Pressure, as an independent thermodynamic parameter, allows intermolecular interactions to be precisely manipulated. Moreover, it drastically alters the electronic structure and bond vibrations of materials and, subsequently, their photophysical properties.^[^ ^22^, ^23^, ^24^, ^25^ ^]^ Recently, the application of pressure using the diamond anvil cell (DAC) technique, in combination with spectroscopic detection, has provided valuable insights into the structure‒property relationship.^[^ ^26^, ^27^, ^28^ ^]^ Herein, we follow the structural changes at the molecular level and correlate the subtle alterations in molecular geometry and intermolecular distances with the photophysical properties of the MR emitters using a combination of high‐pressure in situ optical spectroscopy and theoretical calculations. This combination enables the elucidation of the effect of exciton‒vibration couplings on the FWHM and emission efficiency of the MR emitters in the solid state; this aspect is currently poorly understood. The narrowband MR emitter (BNC, **Figure** **1**a) is prepared by incorporating spherical *o*‐carborane (C_2_B_10_H_12_) as steric hindrance group. The incorporation of icosahedral boron clusters slightly elevates the high‐frequency vibration of the MR framework due to the absence of C═C bonds while simultaneously weakening the molecular interactions. Accordingly, the boron cluster BNC shows a PLQY of 92.9% and small FWHMs of 29 nm at low dopant concentrations. In the crystalline state, the MR‐emitting molecules, which assemble into “isolated dimers, ”clearly quench the PL, with a PLQY of 15.0%. Under low pressure, the PL spectra of BNC crystals exhibit a noticeable broadening, which is primarily attributed to the significant weakening of the PL peak at 536 nm. Interestingly, the low‐frequency and partially high‐frequency vibrations are consistently suppressed from 0.48 to 4.43 GPa, resulting in a reduction in the exciton‒vibration couplings. Consequently, BNC crystals exhibited a noticeable increase in intensity and a slight narrowing of the PL spectra, which are rarely observed in previous reports.^[^ ^29^, ^30^, ^31^ ^]^ At pressures above 4.50 GPa, the molecular distance between the “isolated dimers” decreases, leading to the appearance of excimer fluorescence. The excitonic interactions between closely stacked “isolated dimers” cause fluorescence weakening and PL spectral broadening. These data not only demonstrate the exciton‒vibration coupling behavior of this particular MR emitter at high pressure but also establish that high‐pressure optical techniques provide rich structure‒fluorescence relationships.

## 2 Results and Discussion

### 2.1 Photophysical Properties and Structure of BNC under Ambient Conditions

**Figure 2:**
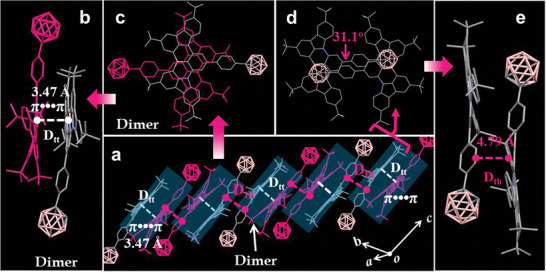
Crystal structures of BNC: a) lateral view of the column arrangement; b,e) front view and c,d) top view of two kinds of antiparallel arrangements.

The photophysical properties of BNC are shown in Figure 1, along with its ultraviolet–visible (UV–vis) absorption and PL spectra. As depicted in Figure 1a, the UV‒Vis spectrum of toluene (1 × 10^−5^ m) exhibited the characteristic MR absorption profile: the intense absorption band at approximately 284 nm corresponded to the local π–π* transitions, and the sharp absorption peak at 471 nm was attributed to the charge transfer transition.^[^ ^32^ ^]^ The BNC solution emitted bright green fluorescence with a PLQY of up to 83.5% (Figure 1b). Its PL spectra demonstrated a clear peak at 492 nm with a very small FWHM of 23 nm. A small FWHM of 22 nm was also observed for the reference luminophore BNH (Figure S4, Supporting Information), which did not contain any spherical *o*‐carborane units. These results showed that the MR characteristics of the B‐N polycyclic framework were not interrupted by icosahedral *o*‐carborane. Moreover, the density functional theory (DFT) calculations were performed at the B3LYP/6‐31G(d,p) level. As depicted in Figure S5 (Supporting Information), BNC and BNH had nearly identical nonbonding molecular orbital distributions, which were consistent with the negligible change in the FWHM of BNC relative to BNH. Interestingly, the PL spectrum showed a mirror image correlation with the absorption, and the observed Stokes shift was only 21 nm. The similarity between the spectra of the crystalline powder, as shown in Figure 1a,b confirmed that the small Stokes shift was caused by the slight structural deformation rather than solvent relaxation.^[^ ^33^ ^]^ Remarkably, the PL spectrum of BNC in the crystalline state had a shoulder peak at approximately 587 nm compared with that in toluene. The transient PL decay curves revealed that the lifetime of the shoulder peak (8.8 ns, Figure 1c) was longer than that of the main peak (1.4 ns). This difference, combined with the molecular packing (Vide infra, **Figure** **2**a), strongly demonstrated that the fluorescence peak at 587 nm was due to the excimer state. In summary, the narrow FWHM and small Stokes shift of BNC were attributed to the unique distribution of the frontier molecular orbitals on the BNC core, which was a result of the nonbonding characteristics of the highly rigid MR framework. This distinctive feature essentially reduced the vibronic coupling between the S_0_ and S_1_ states, as well as the vibrational relaxation at the S_1_ state.

To better understand the origin of the shoulder peak (Vide infra), the BNC crystal was meticulously grown in chloroform through solvent diffusion. The corresponding structures were determined using single‐crystal X‐ray diffraction experiments and belonged to a monoclinic crystal with lattice parameters of *a* = 16.6148(11) Å, *b* = 18.6620(12) Å, *c* = 19.4867(13) Å, *α* = 99.843(2)°, *β* = 113.550 (2)°, and *γ* = 115.882(2)°. In the crystalline state, the BNC monomers had relatively planar structures with a molecular planarity parameter (MPP) of 0.780 Å. The molecules of the BNC crystals adopted two distinct kinds of antiparallel arrangements (Figure 2a): the tail‐to‐tail and head‐to‐tail patterns. For the former, the interplanar π−π distance (*D*_tt_) between the two MR units was 3.47 Å, and the overlapping area was greater than 50% (Figure 2b,c). Moreover, the energy of the interplanar interaction was calculated to be −251 kJ mol^−1^. Therefore, the occurrence of the strong π‒π interaction likely led to the formation of antiparallel dimers. In contrast, one molecule of BNC slid away from its neighbor along the long axis of the molecule, as shown in Figure 2d. The distance between two boron atoms increased from 4.76 Å in the tail‐to‐tail dimer (Figure S6, Supporting Information) to 10.90 Å, resulting in head‐to‐tail packing. Figure 2d shows that the dihedral angle (*θ*_pmr_) between the phenyl ring and its adjacent MR framework was 31.1°. Moreover, the distance *D*_th_ between the phenyl centroid of BNC and the plane of the adjacent molecule reached 4.79 Å (*D*_th_, Figure 2e). These observations indicated the absence of the interplanar π‒π interactions in the head‒to‐tail packing. Therefore, this antiparallel tail‐to‐tail arrangement could be regarded as the isolated and discrete π‒π dimer stacking in the BNC crystals. These results, together with the longer PL lifetime (8.8 ns, Vide supra), showed that the shoulder peak of the BNC crystals at 587 nm was probably due to excimer luminescence of the “isolated dimer” structure. Generally, the “isolated dimer” packing ensures the purity and singularity of the excimer state through exciton localization, thereby preventing the formation of a “dark” state.^[^ ^34^, ^35^ ^]^ Thus, unique dimeric stacking, which promoted discrete excimer formation, was extremely beneficial for achieving high‐efficiency fluorescence. However, in our case, the PLQY of the BNC crystals was 15.0%, which was significantly lower than that of the PMMA film (1‰ wt/wt, PLQY = 92.9%, Figure S7, Supporting Information). This aggregation‐induced PL quenching could be attributed to the high nonradiative rate constant (*k*_nr_ = 1/τ − *k*_r_) of 0.6 ns^−1^. Then, we performed temperature‐dependent PL spectral analysis on the crystalline BNC powder. As the temperature decreased, the PL peak exhibited a blueshift and increased in intensity (Figure S8a, Supporting Information); similar results were previously observed in nonfullerene acceptors.^[^ ^36^ ^]^ Concurrently, the integrated PL intensity monotonically decreased with increasing temperature, as illustrated in Figure S8b (Supporting Information); this enabled the derivation of the corresponding exciton binding energy (*E*_b_) as 34.9 meV using the Arrhenius equation,^[^ ^37^ ^]^ *I*(*T*) = *I*_0_/[1+*A*exp(‐*E*_b_/*k*_b_ *T*)], where *I*_0_ represents the integrated intensity at 0 K, *k*_b_ is the Boltzmann constant and E_b_ denotes the exciton binding energy. Notably, when BNC was doped in PMMA at a weight concentration of 1 wt‰, the resulting film exhibited a large *E*_b_ of 72.4 meV (Figure S9, Supporting Information), nearly double that of the BNC crystals. Clearly, in the doped film, the high *E*_b_ ensured the generation of excitons at room temperature and their high‐rate recombination, and hence, a high PLQY was attained. From the doped film phase to the crystals, the *E*_b_ demonstrated a significant decrease due to the strong polarization effects^[^ ^38^ ^]^ and was slightly greater than the thermal disturbance energy (≈26.0 meV) at room temperature. Therefore, the spontaneous dissociation of excitons at room temperature was effortless, ultimately causing aggregation‐induced PL quenching.

### 2.2 Photophysical Properties of MR BNC at High Pressure

**Figure 3:**
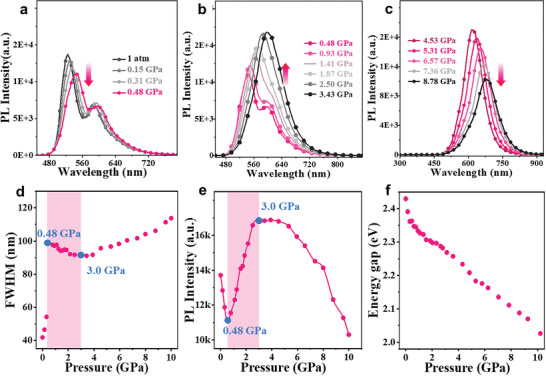
In situ PL spectra of the BNC crystals upon compression from a) 1 atm to 0.48 GPa, b) 0.48 to 3.43 GPa and c) 4.53 to 8.78 GPa. Plots of the d) FWHM, e) PL intensity, and f) energy gap as a function of pressure ranging from 1 atm to 10.21 GPa.

The photophysical properties of BNC changed in response to the application of high pressure, as the molecular packing in the crystals became more compact. As shown in Figure S10a (Supporting Information), piezochromism was visible to the naked eye, with the color of the crystals changing from yellow to deep red as the absorption spectrum shifted toward the red region. The UV‒visible absorption red edge progressively shifted from 518 nm at ambient pressure to 635 nm at 10.20 GPa, resulting in a bathochromic shift of 3557 cm^−1^. Accordingly, the optical energy gap shifted from approximately 2.43 to 2.02 eV (Figure S10b, Supporting Information), indicating that the energy gap of the BNC decreased during compression. When high pressure was released, the UV spectrum reverted back to its original state (Figure S11a, Supporting Information). Interestingly, a good linear relationship was observed between the optical energy gap and external pressure (**Figure** **3**f). Below 0.48 GPa, the PL intensity of the BNC crystals was slightly weakened (Figure 3a,e), in contrast to the sharp broadening of the PL spectrum. When the applied pressure was gradually increased from 0.48 to 3.43 GPa, the PL intensity of BNC significantly and rapidly increased (Figure 3b). As illustrated in Figure 3e, the PL intensity at 3.43 GPa exhibited a remarkable 50% increase relative to that at 0.48 GPa. Moreover, the FWHM of the PL spectra was slightly narrowed, as shown in Figure 3d. Above 4.50 GPa, BNC crystals showed gradually broadened PL spectra and decreased intensity, with a slight redshifted fluorescence (Figure 3c,e). Clearly, under high pressure, the redshift of the absorption and PL spectra clearly indicated a decrease in the energy of the low‐lying excited state. These results indicated that high pressure induced a more compact arrangement of molecules, resulting in increased intermolecular interactions.^[^ ^25^, ^27^, ^28^ ^]^ Upon pressure release, the fluorescence peak returned to its original peak at 536 nm; however, the intensity of the shoulder peak at 587 nm significantly increased (Figure S11b, Supporting Information). As shown in Figure S12 (Supporting Information), the decompressed BNC crystals displayed a single exponential PL decay curve and had a longer lifetime than the original state. Thus, the strength of the interplanar π‒π interactions within the “isolated dimers” did not completely return to their initial state.

### 2.3 Structure of MR BNC at High Pressure

**Figure 4:**
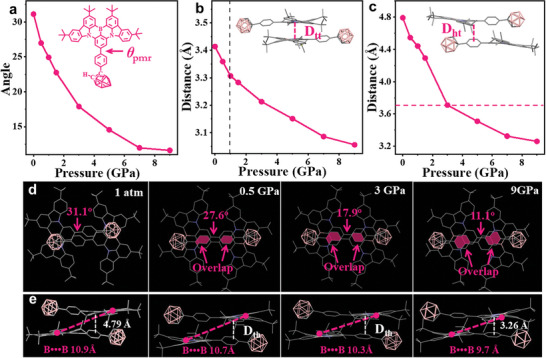
Selected structural parameters: the plots of a) the degree of dihedral angles *θ*_pmr_, interplanar distance b) *D*_tt_ and c) *D*_th_ of the BNC structures versus various pressures from 1 atm −9.00 GPa; additionally, the d) overlap and e) length of the B•••B bond, together with the interplanar distance *D*_th_ of the neighboring molecules showing the head‐to‐tail patterns.

Theoretical calculations of the BNC crystals at various pressures were performed to clarify the abnormal piezochromic fluorescence. Upon compression of the BNC from ambient pressure to 9.00 GPa, the unit cell volume contracted by 722 Å^3^ (−28.0%) (Figure S13a, Supporting Information). The compression of the unit cell was anisotropic, and the *a*‐, *b*‐, and *c*‐axes were shortened by 7.33 Å (−26.0%), 1.51 Å (−6.8%), and 0.926 Å (−4.4%), respectively. The most compressible axis (the *a*‐axis) was aligned with the decrease in the interplanar distance *D*_tt_ between two MR units (Vide infra, **Figure** **4**b), whereas the less compressible *b*‐ and *c*‐axes were coincident with slight slip along the long axis of the molecule. In Figure 4a,d, the molecular coplanarity greatly improved upon compression, as evidenced by a 62.7% decrease in the dihedral angle, *θ*_pmr_, from 31.1° to 11.6°. Furthermore, the distance between neighboring boron atoms slightly decreased, as shown in Figure 4e aligning with the least compressible *c*‐ and *b*‐axes. Consequently, the overlapping area between the phenyl and MR framework moieties considerably expanded (Figure 4d). The interplanar distance *D*_th_ (Figure 4c) also decreased from 1 atm to 9.0 GPa. Notably, the *D*_th_ distance exceeded 3.70 Å at pressures below 3.00 GPa (Figure 4e), which surpassed the range of typical π‒π interaction distance. This result, together with the small overlapping area between the π‐conjugated moieties (Figure 4d), indicated that BNC adopted isolated and discrete π–π dimer stacking at pressures below 3.5 GPa. Upon further compression, the packing of the molecules became denser such that the π‒π dimers changed to a non‐discrete state. Therefore, above 4 GPa, the PL quenching of the BNC crystals was attributed to the intensified nonradiative relaxation caused by long‐range excimers.^[^ ^35^, ^39^ ^]^ Specifically, the non‐discrete stacking of these dimers, caused by the high pressure (>4 GPa), resulted in a low PLQY of excimer fluorescence. The isolated dimer of BNC was extracted from the corresponding unit cell at each pressure for subsequent analysis. As illustrated in Figure 4b, the interplanar distance *D*_tt_ within the isolated dimers consistently decreased with increasing pressure. Interestingly, at pressures up to 0.50 GPa, the distance *D*_tt_ decreased to as small as 3.30 Å, which was rarely observed at ambient pressure. This distance was slightly smaller than the π–π interplanar distance of the excimer equilibrium geometry (*R*_e_ = 3.330 Å).^[^ ^40^ ^]^ Thus, the dimer of BNC likely exhibited a “compressed” ground state characterized by a shortened π–π distance and increased π–π overlap (Vide supra).

### 2.4 Piezochromic Mechanism of BNC

**Figure 5:**
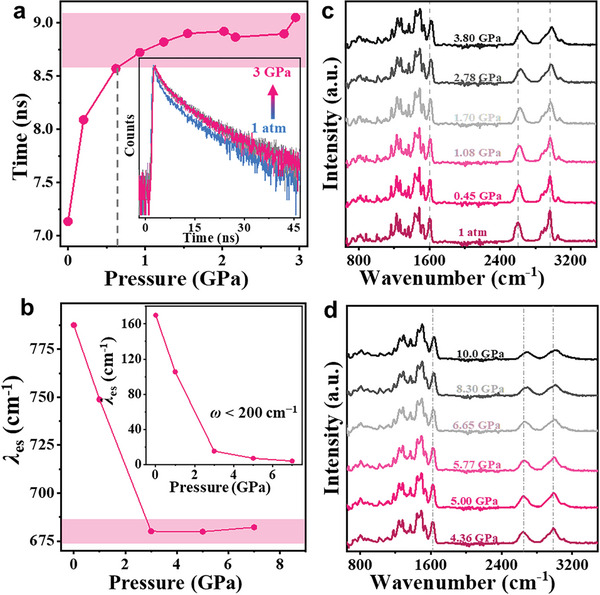
a) PL lifetime and b) calculated reorganization energy as a function of pressure. c,d) Pressure‐dependent IR spectra; the inset in (a) shows transient PL decay curves of the BNC crystals in the pressure range of 1 atm to 3.00 GPa; inset in (b) shows the calculated reorganization energy in the low‐frequency region (*ω* < 200 cm^−1^) as a function of pressure.

The Independent gradient model (IGM) analysis is a valuable tool for visualizing intermolecular interactions in three dimensions (3D). It was utilized to monitor the evolution of these interactions under varying pressures. In the isosurface representation, the blue (red) color indicates the strength of attractive interactions (steric effects), while the green zone signifies π–π interactions. In our case, as depicted in Figure S14 (Supporting Information), the green areas gradually expanded with increasing pressure, demonstrating the presence of strong π–π interactions and π–π overlap within the isolated dimers. Moreover, the pressure‐dependent average PL lifetime τ is calculated by τ = (A_1_τ_1_^2^ + A_2_τ_2_^2^)/(A_1_τ_1_ + A_2_τ_2_). During compression, the PL lifetime of the shoulder peak at 587 nm significantly increased below 0.62 GPa, as shown in **Figure** **5**a. This increase coincided with the PL quenching observed at pressures below 0.48 GPa (Figure 3e). This result further verified that the π–π distance (*D*_tt_) decreased and the π–π overlap increased within the isolated dimers; thus, a “compressed” ground state was formed. Above 1 GPa, the increase in the PL lifetime leveled off. Then, we evaluated the roles of the main excited‐state energy dissipation ways in exciton‒vibration couplings. The strength of the exciton‒vibration couplings was measured by the reorganization energy (*λ*_es_, S_1_→S_0_). Upon compression, from 0 to 3.0 GPa, the *λ*_es_ of BNC in crystalline aggregates monotonically decreased, as depicted in Figure 5b, thus effectively restricting the exciton‒vibration coupling of BNC. With a compression of more than 3 GPa, the value of *λ*_es_ remained close to 680 cm^−1^, suggesting that the exciton‒vibration coupling was not further weakened. In low‐pressure region, the reduction in *λ*_es_ was primarily attributed to the low‐frequency vibrations (*ω* < 200 cm^−1^, inset in Figure 5b) caused by the rotation of the phenyl rings. To further investigate the molecular vibrations, in situ infrared (IR) spectra ranging from 800–3300 cm^−1^ were recorded at high pressures. As depicted in Figure 5c, the relatively prominent peaks at 1528 and 1603 cm^−1^ in the IR spectra at ambient pressure were attributed to the C═C bond stretching vibration of the aromatic ring. With increasing pressure, these peaks representing the C═C bond stretching vibration showed minimal changes in absorption shape and peak position. This observation verified that the decrease in *λ*_es_ for BNC was not influenced by the C═C bond stretching vibration under high pressure. The distinct peaks at 2963.4 and 2603.2 cm^−1^ corresponded to the C─H and B─H bond stretching vibrations of the BNC, respectively. Impressively, the intensity of C─H and B─H stretching vibration modes decreased significantly, while their peak widths increased below 3.0 GPa (Figure 5c,d). These observations suggest that the distances between the C/B atoms and the H atoms of adjacent molecules decreased under high pressure, leading to stronger molecular interactions. Consequently, these interactions restricted the B─H and C─H stretching vibrations, thereby inhibiting the non‐radiative vibrational processes described by Equation (2). However, above 3.0 GPa, the rate of the above change began to slow (Figure 5d), indicating a slight weakening of the exciton‒vibration coupling.

According to the experimental evidence presented, the thorough analysis of the experimental and calculated results led to some reasonable deductions regarding the complicated changes in the PL spectra of the BNC crystals under high pressure as follows: 1) below 0.48 GPa, the pressure caused the contraction of the π‒π distance *D*_tt_ and an increase in the π‒π overlap ratio within dimers to facilitate the enhanced excimer and polarization effects; consequently, E_b_ experienced a slight decrease, leading to a reduction in the PL intensity (Figure 3a); and 2) from 0.48 to 3.0 GPa, the BNC molecules exhibited discrete π‒π dimer stacking; the molecules stacked more tightly within the isolated dimers, resulting in the formation of a “compressed” ground state. Thus, the B─H and C─H stretching vibrations, as well as low‐frequency rotation, were suppressed by high pressure, weakening the exciton‒vibration coupling. This suppression ultimately led to an increase in the PL intensity and spectral narrowing of the BNC crystal (Figure 3b). Upon further compression, the stacking of these dimers produced a non‐discrete state, where long‐range excimers occurred, and PL quenching occurred (Figure 3c).

## 3 Conclusion

In summary, we investigated the piezochromic behavior of a classical MR material and correlated the alterations in intermolecular interactions and molecular vibrations with the photophysical properties. Interestingly, an abnormal phenomenon of pressure‐induced emission enhancement and spectral narrowing was observed in the BNC crystal when the pressure exceeded 0.48 GPa and reached a maximum at 3.0 GPa. Joint in situ experimental and theoretical analyses revealed that the unusual piezochromism was associated with a change in the exciton binding energy and exciton–vibrational coupling. Below 0.48 GPa, the reduction of the *E*_b_ had a greater effect on the relaxation decay of the excited state than the suppression of the exciton–vibrational coupling. Thus, the intensity of PL spectra slightly decreased upon compression. Between approximately 0.48 and 3.0 GPa, the distance *D*_tt_ of the dimers was less than 3.3 Å. Thus, a dimer could be considered a “compressed” emitting unit within this pressure range. The stretching vibrations of B─H and C─H, along with the low‐frequency rotation, were suppressed within the isolated dimer, thereby weakening the exciton‒vibration coupling. Moreover, the *D*_th_ distance exceeded 3.7 Å, indicating that these dimers were isolated and independent and leading to a minor alteration in the polarization effects. the *E*_b_ value of the BNC crystal likely underwent a slight change. Consequently, in the process, the weakening of the exciton–vibrational couplings was dominant; this ultimately resulted in a clear increase in the PL intensity and a slight narrowing of the PL spectra. This study provides an unconventional strategy to better understand the relationships among the intermolecular interactions, molecular vibrations, and luminescence behavior; this understanding is needed for the design of high‐performance narrowband organic light‐emitting materials, according to the desired application.

## 4 Experimental Section

### Preparation of BNC

Both the commercially available B/N skeleton boric acid ester (BCpin) and 1‐(4‐bromophenyl)‐*o*‐carborane were purified through a recrystallization process. BNC were readily prepared via the Suzuki coupling reaction of BCpin with 1‐(4‐bromophenyl)‐o‐carborane (refer to the Supporting Information for the detailed synthetic procedure). The chemical structures of the MR emitters were thoroughly characterized using nuclear magnetic resonance (NMR) spectroscopy (Figures S1 and S2, Supporting Information), mass spectrometry (MS) (Figure S3, Supporting Information), and single‐crystal X‐ray diffraction.

## Conflict of Interest

The authors declare no conflict of interest.

## Supporting information



Supporting Information

## Data Availability

The data that support the findings of this study are available on request from the corresponding author. The data are not publicly available due to privacy or ethical restrictions.
