# Supplementary material for: Pressure‐Induced Emission Enhancement of Multi‐Resonance o‐Carborane Derivatives via Exciton‒Vibration Coupling Suppression
Source: Adv Sci (Weinh). 2025 Jan 27;12(11):2411765. doi: 10.1002/advs.202411765 (PMC11923958; doi:10.1002/advs.202411765)
Supplement: Supplementary file 1 — Supporting Information [file ADVS-12-2411765-s001.docx]

**Pressure-Induced Emission Enhancement of Multi-Resonance o-Carborane Derivatives via Exciton‒Vibration Coupling Suppression**

*Zening Li*^†^*, Qing Zhang*^†^*, Fangxiang Sun, Chunyan Lv, Xinmiao Meng, Yu Hu, Dongqian Xu, Chengjian Li*^*^*, Lei Li***, Kai Wang* *and Yujian Zhang**

† The authors contributed equally to this manuscript.

Prof. Y. Zhang, Z. Li, Y. Hu, and D. Xu

Key Laboratory of the Ministry of Education for Advanced Catalysis Materials, Department of Chemistry, Zhejiang Normal University, Yingbin Road No.688, Jinhua, 321004, People’s Republic of China, E-mail: [sciencezyj@foxmail.com](mailto:sciencezyj@foxmail.com)

Dr.C. Li, Dr. F. Sun and Prof. C. Lv

Department of Materials Chemistry, Huzhou University, East 2nd Ring Rd. No.759, Huzhou, 313000, People’s Republic of China, E-mail: [03307@zjhu.edu.cn](mailto:03307@zjhu.edu.cn)

X. Meng, Prof. L. Li, Prof. K. Wang

School of Physics Science and Information Technology, Liaocheng University, Hunan Road No. 1, Liaocheng, 252000, People’s Republic of China, E-mail: leili@lcu.edu.cn

Keywords: fluorescence, multiple resonance, *o*-carborane, exciton‒vibration couplings

**Experimental and Theoretical Section**

**Atmospheric pressure experiments.** All photophysical measurements, including steady-state and time-resolved PL spectra, were performed on an Edinburgh Instruments FLS1000 with various accessories. ^1^H and ^13^C nuclear magnetic resonance (NMR) spectra were acquired on a Bruker AM500 spectrometer using CDCl_3_ as the solvent and tetramethylsilane (TMS, *δ*=0 ppm) as the internal standard. Atmospheric pressure chemical ionization mass spectrometry (APCI-MS) was performed using a Thermo Fisher® Exactive high-resolution liquid chromatography‒mass spectrometry (LC‒MS) system. The absolute PLQYs of BNC were measured using a Hamamatsu C11347 spectrometer. Powder X-ray diffraction experiments were performed on a Philips X'Pert Pro diffractometer (Netherlands) in the 2θ range of 5–50° at room temperature, with a step size of 0.02° and a scan speed of 2 degrees/min. UV‒visible spectra were recorded on a Shimadzu UV-2600 spectrophotometer (Japan) using quartz cuvettes with a path length of 2 cm. Powder X-ray diffraction data were obtained on a Rigaku Smartlab XE diffractometer using Cu-Kα radiation at 40 kV and 30 mA. The measured 2θ range was from 5° to 50° with a step size of 0.02°, the sample heating rate was 10 K/min, and temperature was maintained for one minute before each test. Single-crystal diffraction data of BNC were collected on a Bruker D8 VENTURE diffractometer using Mo-Kα radiation at an operating voltage of 50 kV, a current of 1.4 mA and a wavelength of 0.71073 Å. Data processing and reduction were performed using APEX3 software, and the crystal data were solved and refined using the OLEX2 1.5 software package and the ShelXL program. The Cambridge Crystallographic Data Centre (CCDC) contains supplementary crystallographic data for BNC under accession number **2285350**.

**High-pressure generation.** High-pressure experiments were performed in a diamond anvil cell (DAC). The samples were loaded into a 120-μm-diameter hole drilled in a T301 stainless-steel gasket. The pressure was calibrated by the fluorescence emission of ruby in the sample chamber. In the experiments for PL and UV–visible absorption experiments of BNC, silicone oil acted as the pressure transmitting medium (PTM) for the high-pressure experiments. The FT-IR experiments were conducted with KBr as PTM.

**In situ high-pressure experiments.** The excitation source used for the photoluminescence (PL) measurements was a 355 nm UV DPSS laser. The high-pressure evolution of the steady-state PL spectra of **BNC** was captured using a modified spectrophotometer (Ocean Optics, QE65 Pro) with a data collection time of 5 s. After passing through a tuneable filter, the laser beam was focused onto the sample using a 10× UV Plan apochromatic objective; the spot size was 20 *μ*m. The lifetime measurements were conducted by Edinburgh FLS1000 photoluminescence spectrometer under the laser excitation at 375 nm. PL micrographs of the samples were captured using a Canon EOS 5D Mark II camera mounted on a Nikon Eclipse TI-U microscope. The camera was set to record photographs under consistent conditions, including exposure time and intensity. In situ high-pressure absorption spectra were obtained using a deuterium-halogen light source (DH-2000-BAL) and recorded with an Ocean Optics QE65 Pro optical fibre spectrometer. The photographs of high-pressure **BNC** were captured using a Canon camera equipped on the light path. In situ high-pressure FT-IR spectra were recorded using a spectrometer equipped with a liquid nitrogen-cooled CCD (SHMADZU, IRTracer-100). All high-pressure experiments were carried out at room temperature.

**Crystal structure compression and ONIOM (QM/MM) simulation**

Based on the BNC crystal obtained at ambient pressure, the unit cells were optimized under various exteral pressures using the Perdew–Burke–Ernzerhof (PBE) functional^1^ with dispersion corrections, embedded in the CASTEP module of the Materials Studio package.^2^ The *k*-point was set to 2 × 2 × 4 during the structural optimization of BNC, and the pressures were set to 0.5, 1.0, 1.5, 3.0, 5.0, 7.0 and 9.0 GPa, respectively. Note that the ambient conditions of NBC crystal experimentally obtained was set to 0 GPa. Based on the optimized crystals, the two-layered ONIOM model with the hybrid quantum mechanics and molecular mechanics (QM/MM) method^3^ was performed to model the pressure influences on the photophysical properties of BNC. In the two-layered ONIOM simulations, the central BNC molecule was set as the high-level QM region and was simulated at the B3LYP/6-31G(d) approach, while all the surroundings were treated as the solid environment and were simulated with the low-level universal force field (UFF) force.^4^ All ONIOM simulations were performed in the Gaussian 16 (A03) software, and all the optimized structures were confirmed as stable structures with no imaginary frequency. Furthermore, the reorganization energies between S_1_ and S_0_ were simulated based on the optimized S_1_ geometries using the DUSHIN software, implemented in the MOMAP package, to evaluate the exciton‒vibration couplings strength of BNC. The independent gradient model (IGM) analysis was adopted to visually evaluate the intermolecular interactions of the interested “isolated dimers” at various external pressures.

**Additional parameters of Eq (2)**

$$k_{nr}\propto V^{2}\left( 4\pi\lambda_{S}\kappa_{B}T \right)^{-0.5}\sum_{j=0}^{\infty} \frac{e^{-s}S^{j}}{j!}\mathrm{ex}p \left[ -\frac{\left( E_{g}-j\hbar\bar{\omega}-\lambda_{s} \right)^{2}}{4\lambda_{s}\kappa_{B}T} \right] \left( 2 \right)$$

For Eq.2, the parameters are as follows: where $\varpi$ is the mean frequency of high-frequency vibration, V is the electronic coupling between the charge-transfer (CT) state and ground state, *λ*_S_ is the structural relaxation energy, *k*_B_ is the Boltzmann constant, T is the Kelvin temperature, *j* is the high-frequency vibrational modes treated quantum mechanically with distinct energy levels (j = {0…n}), (exp(-S)S*^j^*)/j! is the wavefunction overlap of the high-frequency modes between the lowest vibrational CT state (i = 0) and ground state with quantum number j, E_g_ is the energy difference between the ground state and CT state, *ħ* is the Planck constant.

### Synthesis of multiple resonance BNC

**Scheme S1** The synthetic route of **BNC** molecule.

A mixture of 2,5,15,18-Tetrakis(1,1-dimethylethyl)-10-(4,4,5,5-tetramethyl-1,3,2-dioxaborolan-2-yl)indolo [3,2,1-de]indolo[3,2,1:81]-[1,4]benzaza- borino[2,3,4-kl]phenazaborine (BCpin, 0.076 g, 0.10 mmol), 1-(4-bromophenyl)-o-carborane (0.036 g, 0.12 mmol), Pd(PPh_3_)_4_ (0.012 g, 0.01 mmol), and K_2_CO_3_ (0.028 g, 0.2 mmol) were placed in a 50 mL round-bottom flask. The solvent of dimethyl sulfoxide (10 mL) was added, and the mixture was vigorously stirred at 100 ^o^C for 12 h under nitrogen. After cooling, water (30 mL) was added to quench the reaction. The reaction mixture was extracted with ethyl acetate, and the solvent of the organic phase was removed under vacuum. The residue was purified by column chromatography on silica gel with petroleum ether/dichloromethane (4:1) as the eluent to afford a green solid. The desired intermediate compound, **BNC**, was obtained in 70% yield (0.06 g). ^1^H NMR (400 MHz, Chloroform-*d*) *δ* 9.03 (s, 2H), 8.39 (s, 2H), 8.21–8.09 (m, 6H), 7.67–7.58 (m, 2H), 7.56–7.47 (m, 4H), 3.93 (s, 1H), 3.74–1.86 (m, 10H), 1.66 (s, 18H), 1.54 (s, 18H). ^13^C NMR (101 MHz, Chloroform-*d*) *δ* 145.29, 144.66, 144.57, 143.68, 143.19, 143.17, 141.61, 138.07, 132.98, 129.74, 128.15, 127.96, 127.05, 124.32, 123.57, 121.59, 120.77, 117.32, 113.99, 106.61, 60.36, 35.18, 34.80, 32.19, 31.86. ESI-MS m/z: Found 860.6164 [M+H]^+^; ion formula C_54_H_64_B_11_N_2_ requires 860.6164 [M+H]^+^.

**
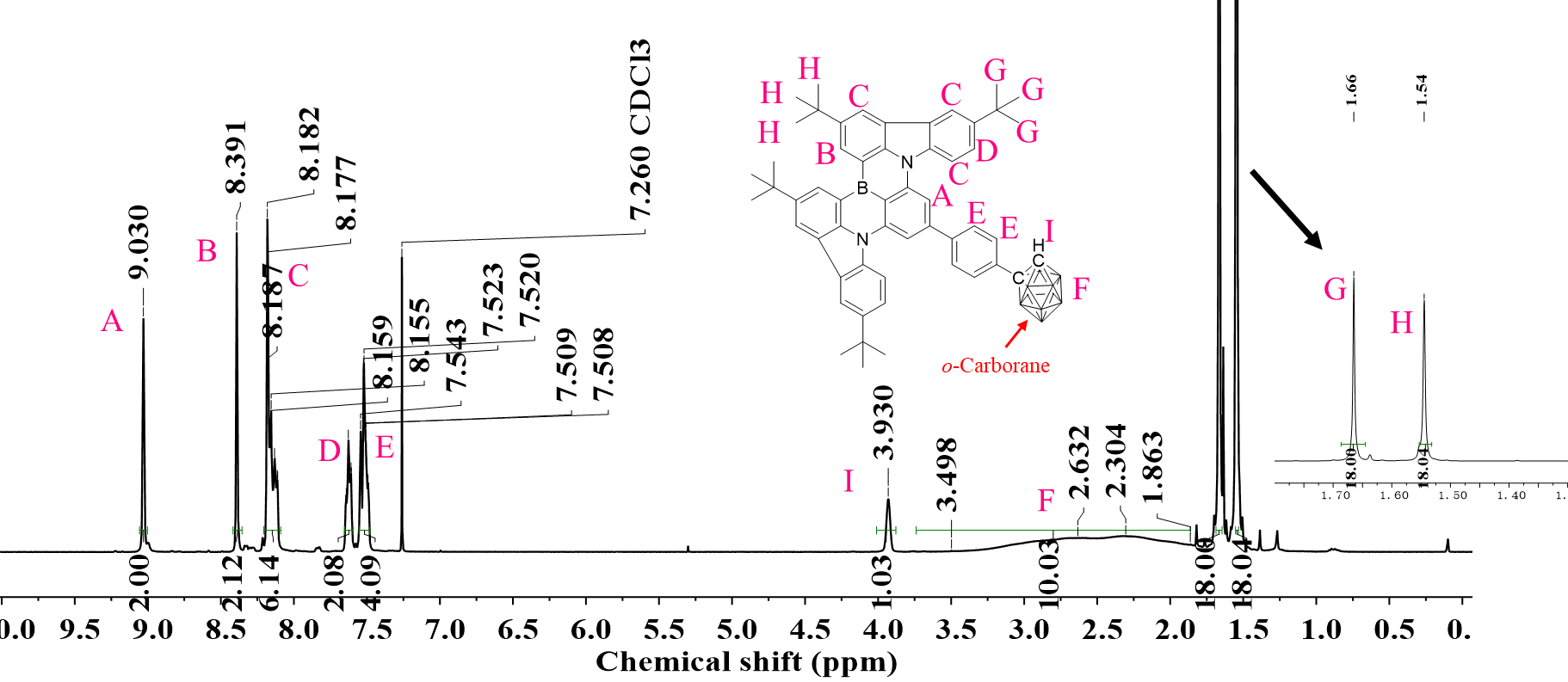
**

**Figure S1**. ^1^H-NMR spectra of **BNC** molecule

**Figure S2** ^13^C NMR spectra of **BNC** molecule


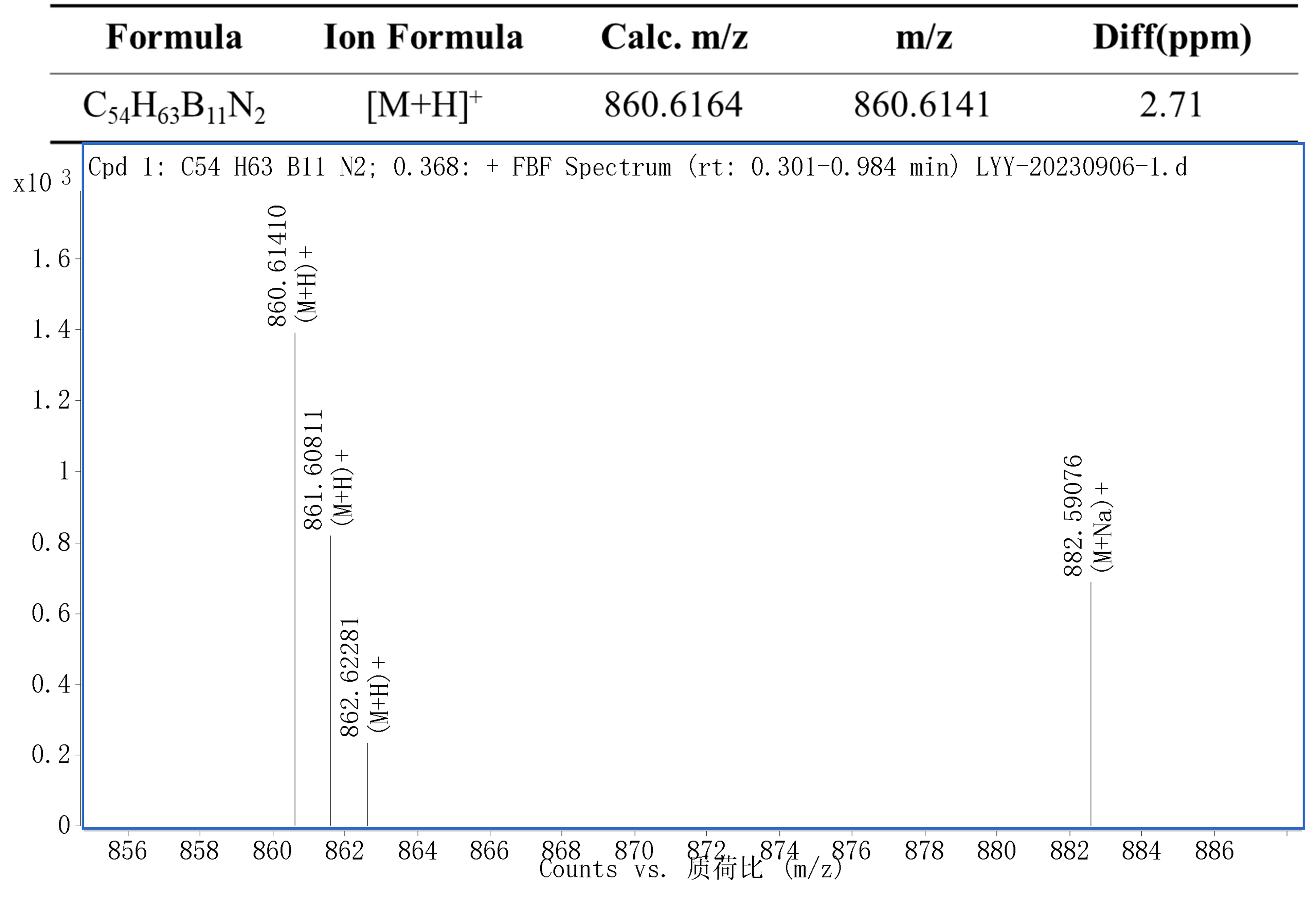


**Figure S3** ESI-MS spectrum of BNC


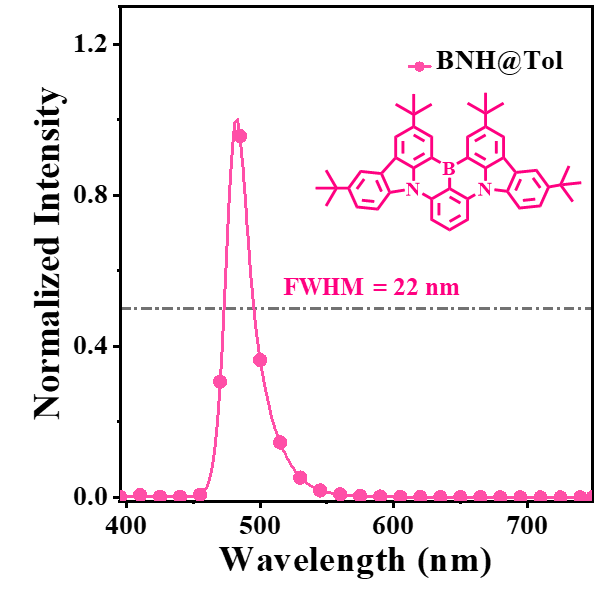


**Figure S4** PL spectra of BNH (10^-5^ M) in toluene solution


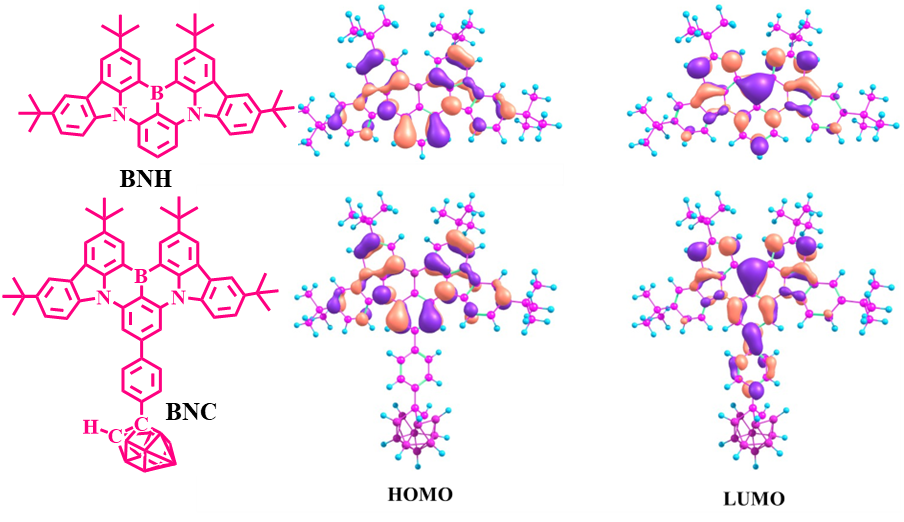


**Figure S5** HOMO and LUMO distributions of BNH and BNC (simulated at the B3LYP/6-31G(d) level by using the Gaussian 16 (A03) package)


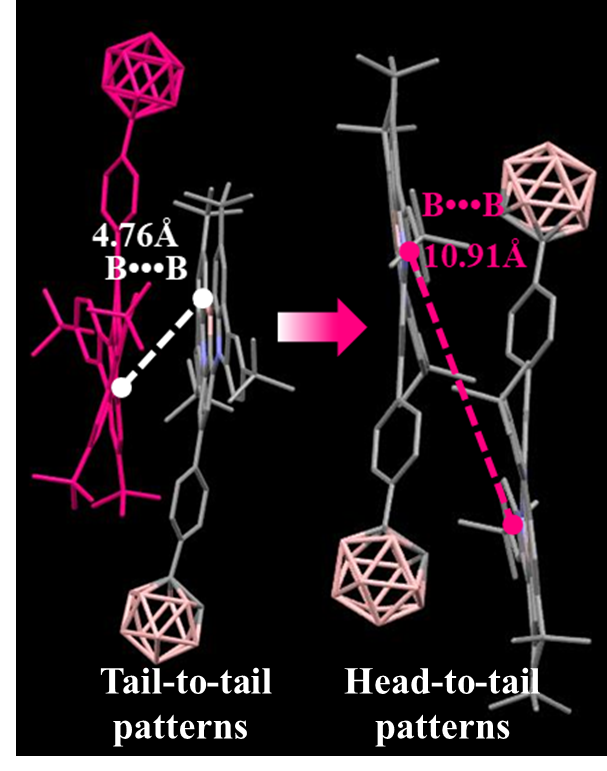


**Figure S6** the The distance between two boron atoms of the tail-to-tail and head-to-tail patterns in the BNC crystal


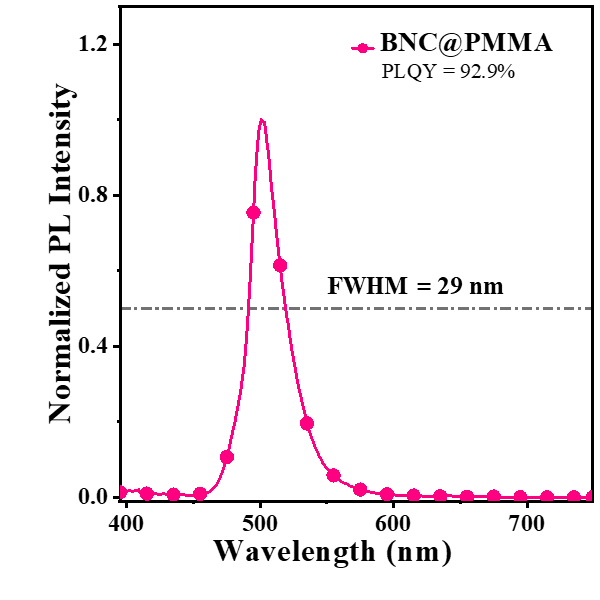


**Figure S7** PL spectra of 1‰ wt/wt BNC-doped PMMA film.


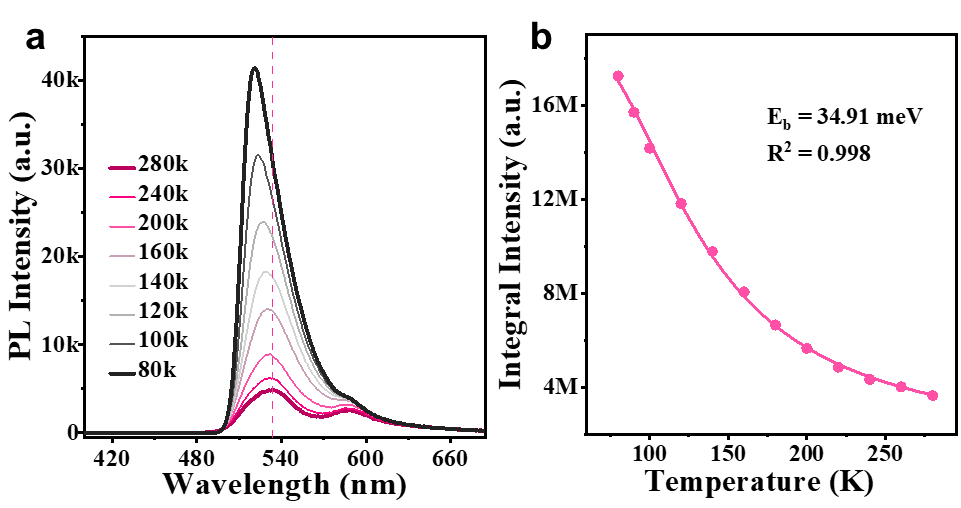


**Figure S8** (a) The PL spectra of **BNC** crystal under different temperature; (b) The fitting graph between temperature and the corresponding spectra integral value by using the Arrhenius equation.


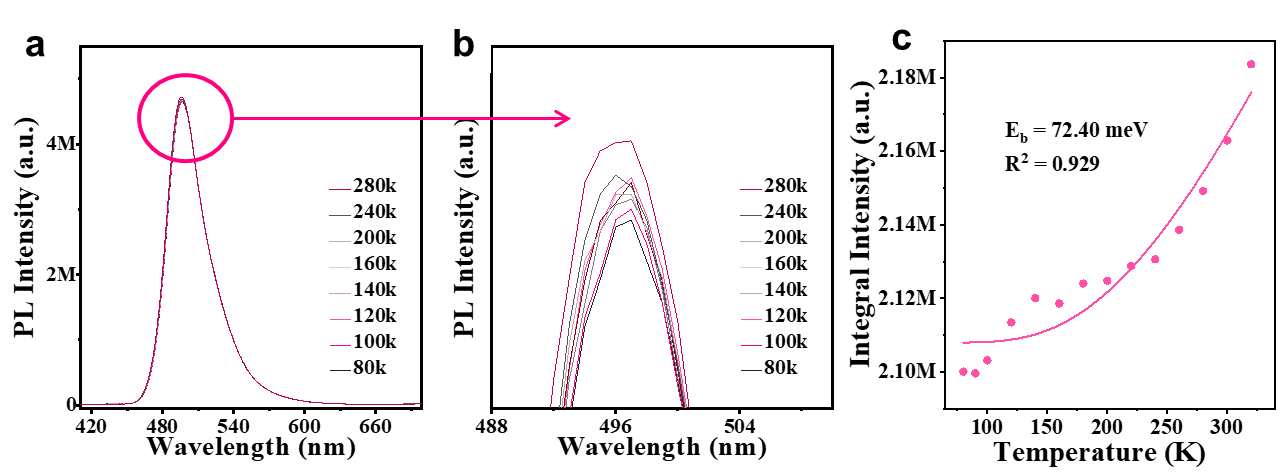


**Figure S9** (a,b) PL spectra of 1 wt‰ BNC-doped PMMA film under different temperature; (c) The fitting graph between temperature and the corresponding spectra integral value by using the Arrhenius equation.


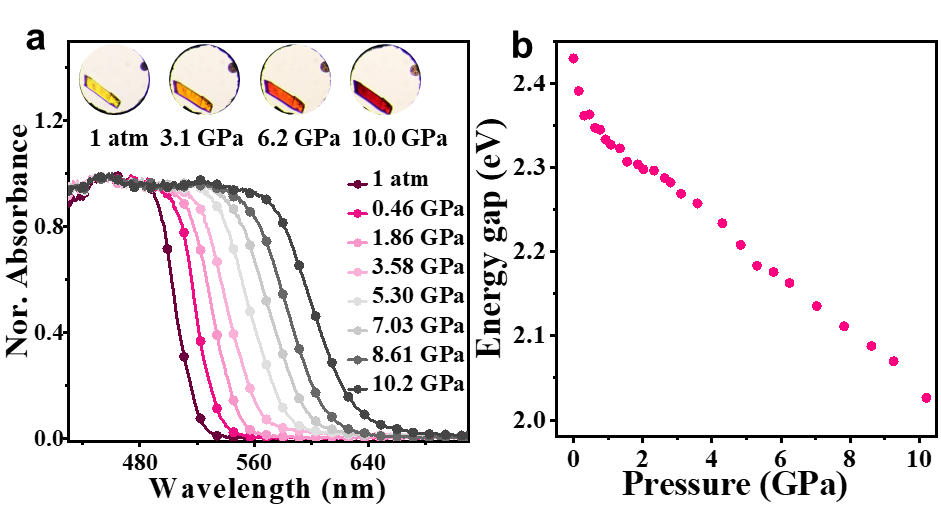


**Figure S10** (a) UV-visible absorption spectrum of BNC crystal upon compression, the inset images show the normal photographs under different pressures; (b) The change of optical energy gap during compression.


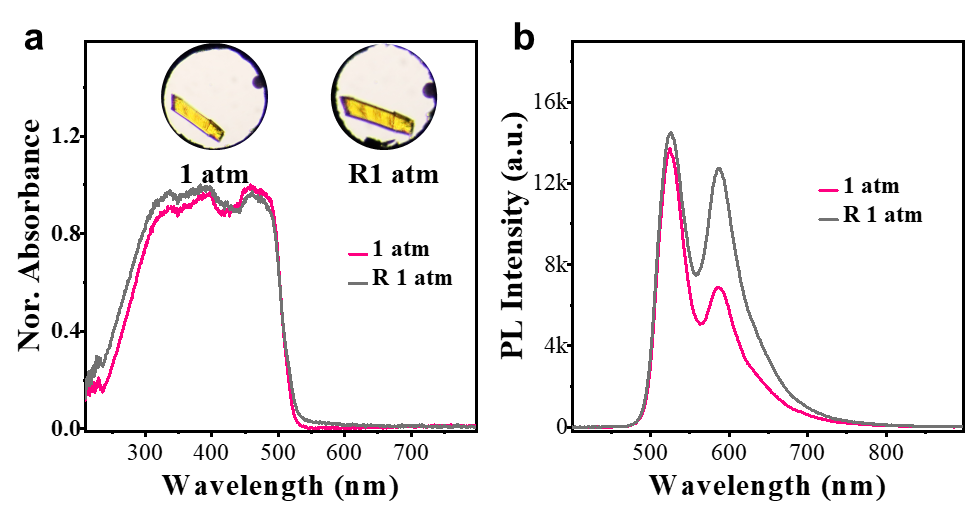


**Figure S11 (a)**the UV-visible absorption spectrum and (b) PL spectra of BNC crystal before compression and after pressure release, the inset images show the photographs at atmospheric pressure.


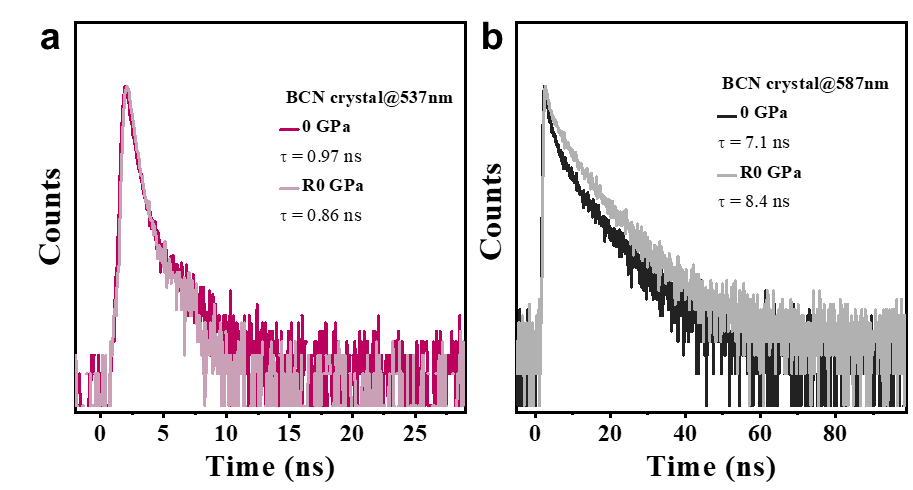


**Figure S12** The transient PL decay curves of **BNC** crystal at the peak of (a) 537 nm and (b) 587 nm before and after compression.


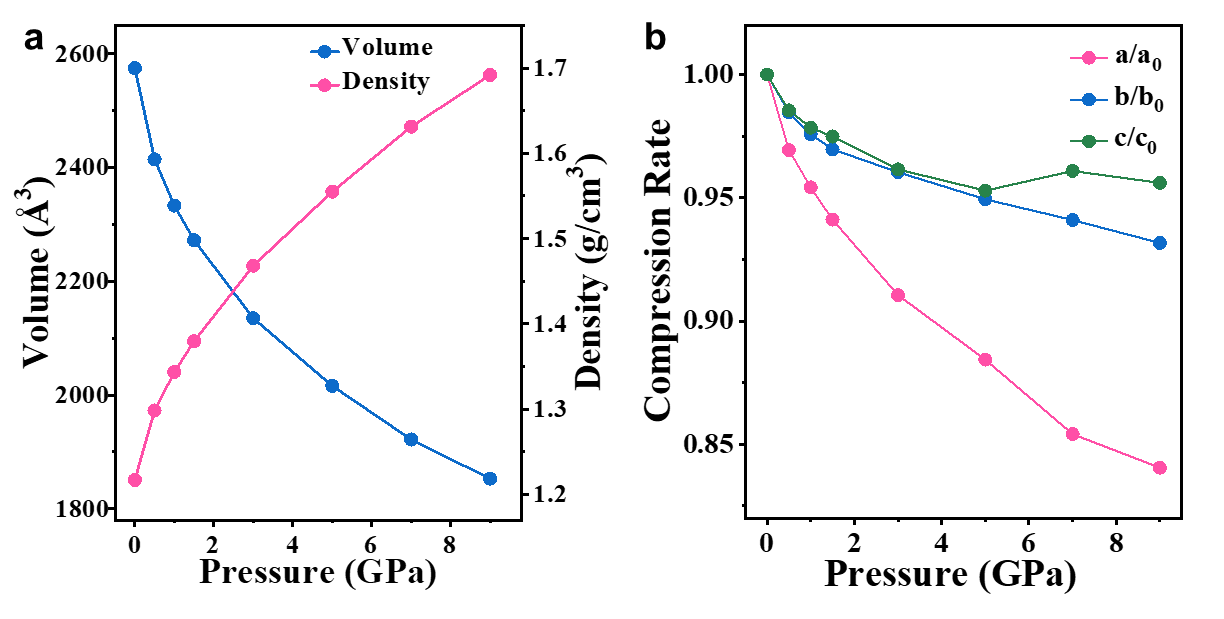


**Figure S13** The changes in BNC crystal unit cell during the compression: (a) volume and density, (b) the rate of length change of the cell axes.


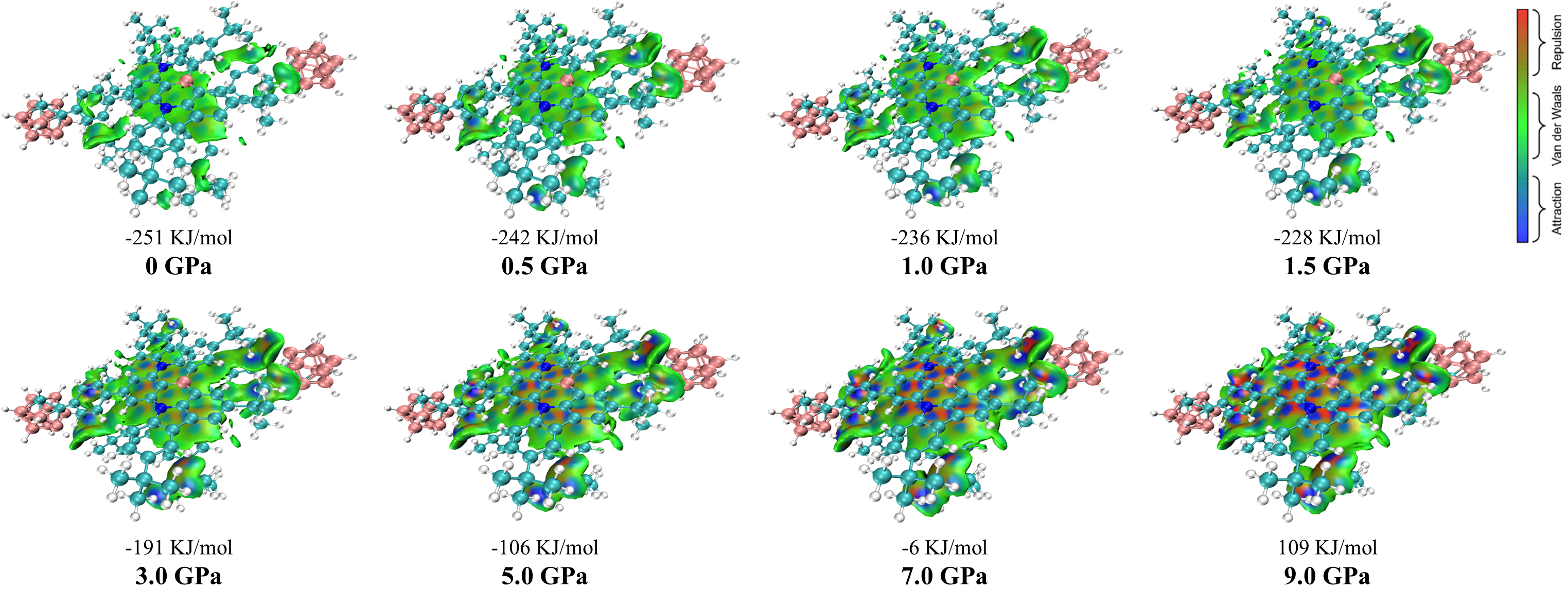


**Figure S14** Visualization of intermolecular interactions for antiparallel dimers in **BNC** crystal under pressures of 0, 0.5, 1.0, 1.5, 3.0, 5.0, 7.0 and 9.0 GPa, respectively, predicted by the IGM method.


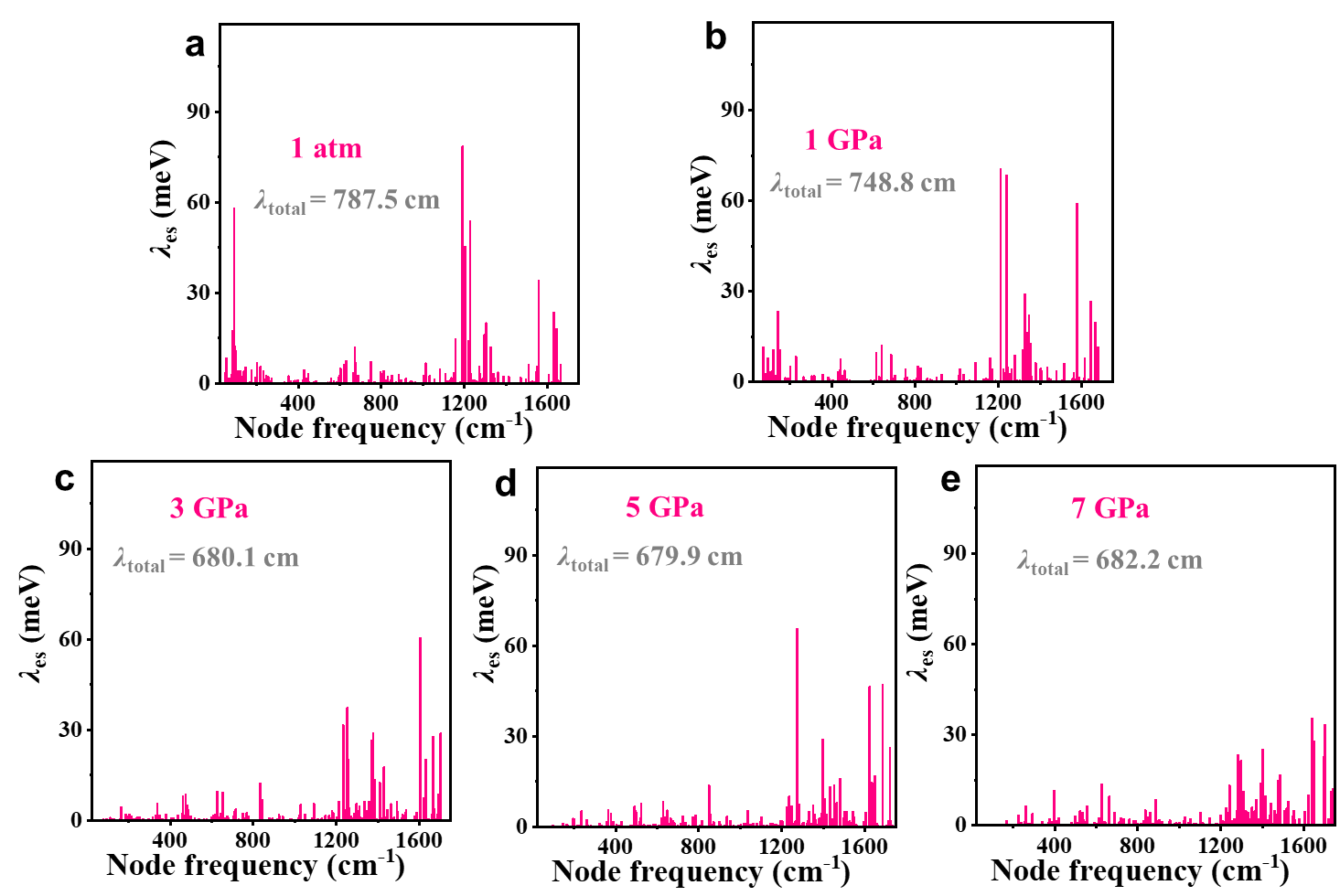


**Figure S15** The calculated total reorganization energy (*λ*_total_) and the contributions of low-, middle- and high-frequency normal modes to *λ*_total_ in **BNC** crystals during compression.

**Table S1** Crystal data and structure refinement for **BNC** crystal.

| Samples | **BNC**  **(CCDC: 2285350)** |
| --- | --- |
| Formula | C54H63B11N2 |
| Mr | 858.97 |
| Temperature(K) | 100 |
| Crystal system | triclinic |
| Space group | P-1 |
| *a*(Å) | 16.6148(11) |
| *b*(Å) | 18.6620(12) |
| *c(*Å) | 19.4867(13) |
| *α*(°) | 99.843(2) |
| *β*(°) | 113.550(2) |
| *γ*(°) | 105.058(2) |
| *V*(Å^3^) | 5084.6(6) |
| *Z* | 4 |
| *D*_calc_ (g/cm^3^) | 1.122 |
| Theta Range(°) | 1.193 – 26.429 |
| F(000) | 1824.0 |
| *h, k, l_max_* | 20, 23, 24 |
| N_ref_ | 20427 |
| T*_min_*, T*_max_* | 0.568, 0.745 |
| Independent reflections | 52983 |
| Goodness-of-fit on F_2_ | 1.024 |
| *R_int_* | 0.0929 |
| *R_1_*[*I*>2σ(*І*)] | 0.0775 |
| *wR_2_*[*I*>2σ(*І*)] | 0.1785 |
| *R_1_*(all data) | 0.1784 |
| *wR_2_*(all data) | 0.2296 |
| S | 1.024 |

*R*_1_ = Σ||*F*_o_| – |*F*_c_||/Σ|*F*_o_|, *wR*_2_ = [Σ*w*(*F*_o_^2^ – *F*_c_^2^)^2^/Σ*w*(*F*_o_^2^)^2^]^1/2^

**References**

1. J. P. Perdew, K. Burke, M. Ernzerhof, *Phys. Rev. Lett.* **1996**, *77*, 3865–3868.
2. S. J. Clark, M. D. Segall, C. J. Pickard, P. J. Hasnip, M. I. J. Probert, K. Refson, M. C. Payne, *Cryst. Mater.* **2005**, *220*, 567–570.
3. L. W. Chung, W. M. C. Sameera, R. Ramozzi, A. J. Page, M. Hatanaka, G. P. Petrova, T. V. Harris, X. Li, Z. Ke, F. Liu, H. B. Li, L. Ding, K. Morokuma, *Chem. Rev.* **2015**, *115*, 5678–5796.
4. A. K. Rappe, C. J. Casewit, K. S. Colwell, W. A. Goddard, W. M. Skiff, *J. Am. Chem. Soc.* **1992**, *114*, 10024–10035.
5. A. K. Rappe, W. A. Goddard, *J. Phys. Chem.* **1991**, *95*, 3358–3363.
